# Understanding Pandemic Solidarity: Mutual Support During the First COVID-19 Lockdown in the United Kingdom

**DOI:** 10.1093/phe/phad024

**Published:** 2023-12-09

**Authors:** Stephanie Johnson, Stephen Roberts, Sarah Hayes, Amelia Fiske, Federica Lucivero, Stuart McLennan, Amicia Phillips, Gabrielle Samuel, Barbara Prainsack

**Affiliations:** Ethox Centre, Nuffield Department of Population Health, University of Oxford, Oxford, UK; Institute for Global Health, University College London (UCL), London, UK; Department of Health Policy, London School of Economics and Political Science (LSE), London, UK; Vienna School of International Studies, Diplomatic Academy Vienna, Vienna, Austria; Institute of History and Ethics in Medicine, Department of Clinical Medicine, TUM School of Medicine and Health, Technical University of Munich, Munich, Germany; Ethox Centre, Nuffield Department of Population Health, University of Oxford, Oxford, UK; Institute of History and Ethics in Medicine, Department of Clinical Medicine, TUM School of Medicine and Health, Technical University of Munich, Munich, Germany; Centre for Biomedical Ethics and Law, Department of Public Health and Primary Care, KU Leuven, Leuven, Belgium; Department of Global Health and Social Medicine, King’s College London, Bush House, The Strand, London, UK; Department of Political Science, University of Vienna, Neues Institutsgebäude, Universitätsstraße 7, 1010 Vienna, Austria

## Abstract

Throughout the COVID-19 pandemic, the concept of solidarity has been invoked frequently. Much interest has centred around how citizens and communities support one another during times of uncertainty. Yet, empirical research which accounts and understands citizen’s views on pandemic solidarity, or their actual practices has remained limited. Drawing upon the analysis of data from 35 qualitative interviews, this article investigates how residents in England and Scotland enacted, understood, or criticised (the lack of) solidarity during the first national lockdown in the United Kingdom in April 2020—at a time when media celebrated solidarity as being at an all-time high. It finds that although solidarity was practiced by some people, the perceived lack of solidarity was just as pronounced. We conclude that despite frequent mobilisations of solidarity by policy makers and other public actors, actual practices of solidarity are poorly understood—despite the importance of solidarity for public health and policy.

## Teaser

This article investigates how residents in England and Scotland enacted, understood or criticised (the lack of) solidarity during the first national lockdown in the United Kingdom in April 2020—at a time when media celebrated solidarity as being at an all-time high. It finds that although solidarity was enacted by some people, the perceived lack of solidarity was just as pronounced. We conclude that despite frequent mobilisations of solidarity by policy makers and other public actors, actual practices of solidarity are poorly understood—despite the importance of solidarity for public health and policy.

## Introduction

The concept of solidarity was invoked extensively during the initial period of the COVID-19 pandemic. In the United Kingdom, it was appealed to when citizens were asked to restrict their movements and contacts to ‘flatten the curve’ of infections, to wear masks (Rothstein, 2020; Silva et al., 2021) or get vaccinated (Ewuoso et al, 2022). In the first months of the pandemic, public and social media were also rife with reports of people taking care of each other and of communities coming closer together.

Against this backdrop at the onset of COVID-19 in the United Kingdom, this article asks: How did participants understand solidarity, and what solidaristic practices did people engage or experience in during the first national lockdown in April 2020? In contrast to explorations of pandemic solidarity so far, which were mostly theoretical, we discuss solidarity based on data on people’s experiences and practices. We explore and consider how people’s actions during the pandemic map onto academic and policy understandings of solidarity and identify differences and gaps. We also reflect on how manifestations of solidarity within real world settings, and observed from what people think, say and do can help to both advance theoretical understandings of solidarity and be better supported and fostered in relation to ongoing and future public health concerns.

## Background: The Pandemic in the United Kingdom

In the United Kingdom, the first two cases of COVID-19 were reported in York on 30 January 2020 (BBC, 2020), on the same day in which the World Health Organization (WHO) declared a public health emergency of international concern (PHEIC) in response to the rapid spread of the virus (WHO, 2020). This was followed by a range of public health and response measures to limit the spread of the virus before the WHO characterised the outbreak as a pandemic on 11 March 2020 (WHO, 2020). On 23 March 2020, the first national lockdown was declared in the United Kingdom, in which people were ‘ordered to stay at home’ to ‘save lives and protect the NHS’ (UK Government, 2020).

Amid the onset of the first wave of COVID-19 in the United Kingdom, references to solidarity emerged as communities grappled with the sudden uncertainty brought forth by the pandemic and the imposition of the first national lockdown. Early in the national lockdown, the UK Government partnered with the newspaper industry to promote the ‘All in, all together’ campaign, a three-month advertising campaign which emphasised the collective risk and experience of the pandemic (Society of Editors, 2020). Messaging to the public during this time included ‘Stay at home for the NHS, your family, your neighbours, your nation, the world and life itself’ and was featured as a cover wrap and online homepage takeover on all regional and national daily newspapers (Society of Editors, 2020). Other examples included the assembling of local community support and mutual aid groups to provide support and presence to many vulnerable community members including the elderly and immunocompromised individuals who had been advised to shelter indoors both before and throughout the period of lockdown. Throughout the first national lockdown, one of the best-known of these organisations developed for community support resources across the United Kingdom was *COVID-19 Mutual Aid UK,* an umbrella organisation of support and aid groups operating at community level which delivered various essential goods and pastoral care to sheltering community members, including food, medicines, and check-in calls (COVID-19 Mutual Aid UK, 2022).

During the early coronavirus crisis in the United Kingdom, as highlighted by Wood and Skeggs (2020: 641), people across the country also engaged in expressions of gratitude—and arguably also solidarity—toward the National Health Service (NHS) and front-line workers in a range of ways: ranging from adorning windows with pictures of children’s rainbows, to lighting up buildings in the blue and white livery of the NHS, to farmers ploughing NHS signs into their fields. Amid an upsurge of appreciation and awareness of front-line health workers and the NHS, the most widely discussed and documented show of solidarity at the time of the first national lockdown arguably was ‘Clap for Our Carers’, which took place every Thursday evening at 20:00 across the country, between 26 March and 28 May 2020 and in which communities joined in clapping from their doorsteps and windows in a simultaneous, weekly demonstration of support for the NHS and frontline health workers during a period of mounting infections, health systems strain and deaths from the first wave of COVID-19.

While these events during the first national lockdown in the United Kingdom suggested the rise of solidaristic practices among communities during the early months of the COVID-19 pandemic, these examples also justify the need for deeper and more nuanced investigations for practices and perspectives of solidarity during periods of crisis. Specifically, the above and often discussed examples of solidarity were focused on large-scale acts of solidarity during this time which were extensively captured and reported by media. While national newspaper campaigns, mutual aid groups and ‘Clap for Our Carers’ were widely referenced and visible during this period, significantly less investigation and analysis has been attributed to individual perspectives and understandings of solidaristic practices in the United Kingdom during the onset of the pandemic, which thus served as a critical point of investigation and rationale in the research aim and results of this study.

## What Does Scholarship Have to Offer?

What is the status of scholarship on solidarity relevant to pandemics to date? Within the wide literature on solidarity, the concept is used in different contexts, to support different goals, and with many different meanings (for overviews of the—mostly English language—literature, see Prainsack and Buyx, 2017). Some authors see it as a moral ideal (e.g. Rorty, 1989; Komter, 2005), as a ‘natural’ or even strategic characteristic of groups or societies (e.g. Hechter, 1987), a political ideal (Mason, 2000; Brunkhorst, 2005; Scholz, 2008; Wilde, 2013), or an economic vision (e.g. Allard et al., 2008). Some see it as practice with sensory and emotional properties (e.g. Dean 1996; Prainsack and Buyx, 2017; Atuire and Hassoun 2023). Dawson and Verweij (2012) propose a concept of solidarity in which a form of ‘greater good’ should be the aim of every individual that is part of a society. Dawsons’ and Verweij’s concept distinguishes between two forms of solidarity: *Rational solidarity* can be observed in the context of collective actions being implemented by relevant government authorities, for example, to mitigate a nationwide or global threat—such as a pandemic situation. *Constitutive solidarity* differs from the first kind in the way that it does not arise from a cost-benefit analysis (2), but from a set of norms and values that is predefined by a closer social group (community) and is a decisive reason for people who identify themselves with this group to enact solidarity. Sticking with the example of a pandemic or a similar health crisis, constitutive solidarity might involve certain practices that can help to protect or restore the health of community members, such as voluntary social distancing (4) or taking care of sick neighbours. Fundamental for both forms of solidarity is the assumption that people are willing to act as a collective and acknowledge that sharing risks and burdens is crucial to overcome threats to individuals as well as to communities and entire societies (Verweij 2015).

Despite these differences, virtually all conceptualisations of solidarity have three things in common: First, they refer to some kind of support, such as people standing up with, besides, or for each other (e.g. Brunkhorst, 2005; Dawson and Jennings, 2012) or for non-human others (e.g. Rock and Degeling 2015). Second, those providing support have something in common with those who receive the support: A shared goal, a common characteristic, or a common threat. Despite the many ways in which actors are different, within solidarity, it is the similarities, and not the differences, that give rise to action (Calhoun, 2002; Prainsack and Buyx, 2011, 2017; Atuire and Hassoun 2023). Third, solidarity is typically seen not as an isolated, one-off, interaction, but as part of a social or political fabric (e.g. Sternø, 2005; Molm et al., 2007). Jürgen Habermas’ (1990: 244) description of solidarity as ‘the reverse side of justice’ captures the nature of solidarity as the ‘glue’ between the bricks that make the architecture of our political and social institutions (see also Scholz, 2008). Within this architecture, solidarity is that which cannot be prescribed, but what people do on their own initiative and will.

In sum, most authors treat solidarity as a prosocial notion that (i) refers to some kind of support that people give to others, (ii) with whom they consider themselves as connected in some way or another and that (iii) is more than a one-off interaction between two individuals but becomes institutionalised, either formally or informally. This also means that whereas solidarity need not be exclusively directed at supporting the most vulnerable, it will regularly be vulnerable people and groups that are intended to benefit most immediately from solidaristic practices and institutions. Here, practices of solidarity regularly shade into other practices that are often subsumed under the label of altruism. Yet at the conceptual level, some important differences between solidarity and altruism remain. First, if altruism is behaviour ‘motivated by a desire to benefit someone other than oneself for that person’s sake’ (Kraut, 2020: 1), then solidarity is a wider concept than altruism. Solidarity does not merely focus on the intention to benefit someone other than ourselves, but also on the reason why we want to do it: namely because there is something that we feel we have in common with those that we set out to support. Second, while altruism can refer to specific instances of practice, it can also refer to a general disposition of a person towards others: We would call someone an altruistic person if they regularly put other people’s interests before their own (Rushton et al., 1981; Bierhoff and Rohmann, 2004. Solidarity, in contrast, does not exist at this general level; it makes no sense to call someone a ‘solidaristic person’. Solidarity is always linked to concrete practices within a community of people who are sharing certain things in common, or to policies and institutions that are designed to harness and support such practices. Third, while it is possible to conceive instances of altruism that are ‘pure’ in the sense that they contain no other motivations than that to benefit others (Ferguson et al., 2012; Ottoni-Wilhelm et al., 2017), by the vast majority of scholarship, solidarity is treated as a relational concept that considers both the givers and the receivers of solidarity as being changed in the process. Also because solidarity is based upon the sense on the side of those practicing solidarity that they share something in common with those who they seek to support, it is impossible, in the case of solidarity, to speak of ‘pure’ other-directedness. This is also one of the points that have been raised in critique of Titmuss’ (1970) seminal work on altruistic blood donation as a ‘gift relationship’ conceptualised as purely focused on benefitting others (eg Pinker 2006). As Parry (2008) argues, the assumed dichotomy between altruism and self-interest in Titmuss’ work does not adequately reflect the nuances in actual practices in this field; they include more than only pure altruism or gain-oriented commodification. Indeed, many empirical studies with donors of biological material indicate that in giving typically contains both altruistic and self-interested elements, which are inseparable from each other. As Shaw (2007: 293) put it, donors ‘moral identities as ethical subjects are created in the donative process’.

An aspect addressed in some scholarship on solidarity that is particularly relevant for our own empirical work is the assumption that solidarity is either getting stronger during crises, or that it is particularly important during crises. Waldby and Mitchell (2006) for example, discuss the relevance of Titmuss’ work on the gift relationship for solidarity in the context of the acute crisis of a war: ‘Giving blood to the troops was a way to express solidarity and improve morale in the anxious conditions of world war’ (Waldby and Robert 2006: 3). This does not only apply to the world wars of the 20th century but also to the terrorist attacks of the late 20th and early 21st (see also Starr [1998: 154]), in which blood donation has become symbol of a new social contract in and beyond crises.

Regarding solidarity in pandemics, only very little conceptual work was published in English prior to COVID-19. Pre-COVID-19 publications discuss solidarity as a practice of, and in support of, healthcare workers during pandemics (e.g. Brody and Avery 2009).^1^Krishnamurthy (2013: 129), discussing Canada’s response to the H1N1 pandemic and the failings of these responses regarding Aboriginal communities in Canada, referred to political solidarity as a relational concept in the sense ‘that citizens of a shared state can be said to stand in such a relation when they have attitudes of collective identification, mutual respect, mutual trust, loyalty and mutual support toward one another’. Lundgren (2016) discussed how the Swedish governmental response to the H1N1 pandemic framed vaccination and bodily practice with the goal to reach herd immunity in terms of solidarity, which however started to suffer cracks when side-effects of the vaccine Pandremix became visible. In another paper, Lundgren (2017: 22), discussed different ways of ‘arguing for solidarity, herd immunity and social justice and claims for culpability of the state’ based on interviews with two communities (the National Pandemic Group and the Narcolepsy Association) during the H1N1 influenza pandemic in Sweden. In 2011, Prainsack and Buyx explored the applicability of the notion of solidarity to pandemic measures, and concluded that it is not well suited, neither to serve as a guiding concept, nor as an analytic lens in times of a pandemic when it comes to interpersonal practices (Prainsack and Buyx, 2011). They argued that ‘it is unreasonable to expect that entire populations—where risks and stakes are very unevenly distributed—will accept the costs of containing pandemics out of solidarity with each other’ (Prainsack and Buyx, 2011: 68). An empirical study from 2009, investigating Public Deliberation About Social Distancing Measures in a Pandemic, had indeed found that ‘social distancing measures may be challenging to implement and sustain due to strains on family resources and lack of trust in government’ (Baum, Jacobson, and Goold, 2009: 4). A 2016 study found that in a pandemic participants expected a low personal infection risk, which resulted in a low willingness to get vaccinated (Determann et al., 2016). Finally, solidarity at the level of relationships between countries (and other global actors) during pandemics received attention in the context of debates on global public health, and global bioethics (e.g. Pang, 2016).

The COVID-19 pandemic, therefore, presents further opportunity and scope to analyse the robustness of our conceptual understandings of solidarity as applied and tested within real-world settings, and how such understandings can be engaged, mapped and leveraged for better practices and development of policy during health emergencies.

## Methods

### Study Design

We conducted 35 semi-structured qualitative interviews with residents of England and Scotland, to specifically examine and understand practices of solidarity within the United Kingdom during the first national lockdown in April 2020. All members of the research team were part of a larger and ongoing qualitative, longitudinal and multinational consortium ‘Solidarity in times of pandemics’ (SolPan). The SolPan consortium comprises of ten European countries (Austria, Belgium, Germany, France, Ireland, Italy, The Netherlands, Switzerland, the United Kingdom and Portugal, which joined in 2021). At the beginning of the COVID-19 pandemic, SolPan set out to explore peoples’ experiences during this global health crisis, with particular attention to how people described practices relating to solidarity. The working definition of solidarity used for this purpose was deliberately broad. In line with the three core elements of solidarity distilled from (English language) literature on solidarity as presented above, we qualified anything as relevant to solidarity that referred to some kind of support that people give to (or receive from) others with whom they consider themselves as connected in some way or another, and/or to institutionalised forms of such support. The codes that we used for this purpose included, for example, ‘supporting/not supporting practices’, with sub-categories pertaining to providing support for family, for other people outside the family, to lack of support, or to offers of support accepted or not accepted. For full details of the design and methodology of the SolPan Project see Wagenaar et al. 2022; for the study interview guide and code book, see SolPan Consortium (2021).

### Participant Selection

Participants were initially recruited for this study via convenience sampling methods. Information and contact details about the study were signposted via online advertisement on participating university websites and social media networks. Chain-referral sampling methods were additionally utilised as participants joined the study and suggested further contacts for participation and contribution within the study. To enable a maximum variety of perspectives, participants were recruited with attention to a range of different demographics, including age, gender, income, household structure, geographic area, education and employment. Demographics of interview participants are reported in Table 1.

**Table 1.** Self-reported demographic characteristics of participants

Characteristic	Number of participants n/N(%)
**Gender**	
Male	20/35 (57)
Female	14/35 (40)
**Age**	
18-30	6/35 (17)
31-45	11/35 (31)
46-60	11/35 (31)
61-70	5/35 (14)
70 plus	2/35 (6)
**Employment status**	
Employed (long-term contract)	17/35 (49)
Employed (short-term contract)	2/35 (16)
Self-employed	5/35 (14)
Unemployed	5/35 (14)
Retired	5/35 (14)
Other	2/35 (16)
**Highest level of education**	
Less than 10 years	2/35 (6)
10-14 years (e.g. high school diploma)	10/35 (29)
Higher Education	23/35 (66)

### Data Collection

Interviews were conducted between 6 April and 30 April 2020, during the time of the first national lockdown. A collectively developed interview guide was used (SolPan Consortium, 2021). Rather than asking participants about solidarity explicitly, we asked them about the challenges they and other people were facing during the initial stages of pandemic, how they responded to them, and what experiences they had with other people in this context (SolPan Research Commons, 2021). From their responses we inferred how people’s practices, needs, and experiences mapped against the presented definitions of solidarity. Interviews ranged from 30 min to 1 hr 5 min and were all conducted in English. Interviews were recorded digitally or using a GDPR-compliant video chat recorder. Only audio material was stored. All interviews were transcribed and subsequently pseudonymized.

### Data Analysis

Interview transcripts were analysed iteratively and coded by all authors, utilising a pre-generated coding scheme (SolPan Consortium, 2021) with the assistance of the Atlas.ti software. The coding scheme was developed by the ‘SolPan analysis team’, consisting of one representative from each SolPan country group with expertise in qualitative data analysis. Initial descriptive codes were generated by each member of the analysis team. A code book was then generated over rounds of inductive analysis of the data set, and through memoing and discussing emergent findings between members of the analysis team (see Wagenaar *et al.* 2022). All country datasets were then coded using the code book.

For this work, the UK team within SolPan aimed to conduct an analysis of the data set that was attentive to any reports of, or references to, practices that could—according to the key elements of solidarity distilled from the literature above—be qualified as solidaristic, either at the level of individuals, groups or institutions, as well as the perceived lack thereof. Specifically, the researchers aimed to identify data relevant to answering the following research questions:

1. What solidaristic practices do people describe across the three tiers?
  a) Identify the explicit or implicit similarities and costs
  b) Includes identifying self-practices or observed practices (by others e.g. community groups)
2. What practices do people describe that were NOT solidaristic, for example, panic buying
3. What motivates these solidaristic practices?

The three tiers of solidarity were defined as per Buyx and Prainsack:

**Table:** 

**Tier 1: interpersonal solidarity**: The first tier of solidarity is that which is practiced between individual people.
**Tier 2: Group solidarity:** On this tier, solidarity comprises manifestations of a shared commitment to carry costs to assist others with whom people consider themselves bound together through at least
one similarity in a relevant respect (e.g. a shared situation, characteristic, or cause).
**Tier 3: Contractual, Legal or Administrative Norms:** The third tier comprises solidarity that has ‘solidified’ into binding norms and institutions. This is the only form of solidarity that is not always voluntary.

Participants did not need to label these examples themselves as explicitly ‘solidaristic’, as this would have artificially limited and restricted the data set to the use of the term solidarity. Authors were also encouraged to identify data that contradicted the framing of solidarity presented here. For example, if data were identified that undermined the framing of three tiers of solidarity, authors were encouraged to include this in analysis. Initially, each team member reviewed a subset of interviews and extracted data that they determined to be relevant to the research questions. Relevant text passages were extracted using Atlas.ti and analysed inductively, looking for emerging themes and relationships in responses relating to solidarity. Each interview transcript was checked, discussed, and contrasted by a second researcher for consistency. Subsequently, an iterative process of development, discussion and reflection among the authors produced final higher order themes.

Our key findings are structured in three parts. First, we describe our interpretation of the solidaristic practices which were observed and understood by our interviewees during the onset of the first national lockdown in the United Kingdom. Here, we provide in verbatim a range of factors, experiences, and practices which participants inferred as solidaristic behaviour during this period of crisis in the United Kingdom. Second, and in also drawing important focus towards contrasts of solidaristic practices, we describe and present the underlying rationales and perspectives of participants which were associated with non-solidarity during this period, highlighting three key areas in which non-solidaristic behaviour and experiences can be grouped from the data. Third, and perhaps most critically, we also present and detail how many participants’ references to a lack of solidaristic practices during the first national lockdown was also intertwined with emergent expressions or hopes for the support or enacting of institutional forms of solidarity (including political, economic, and social support), and draw important focus for the potential for instutionalised practices of solidarity to mitigate against the collective impacts of future national or global crises.

We then further discuss how these key findings map onto or challenge existing understandings and conceptualisations of solidarity, particularly during public health emergencies in present literature and scholarship. In the concluding section of this article, we reflect upon the present gaps and challenges in conceptualising solidarity as seen through the first COVID-19 lockdown in the United Kingdom and identify the how new conceptual insights produced in this work, while also drawing attention and new discussion to the salience of institutionalised practices of solidarity for increasing collective security and support during crises.

### Ethics

The study was approved by the University of Vienna Ethics Committee Reference Number: 00544. Consent from all participants was obtained orally directly before the interview.

## Results

### Limitations

This study also acknowledges several limitations. First, we note that people with a higher education background are considerably overrepresented in our sample (66%, instead of the national average of 34% as estimated by the Office for National Statistics). Given this overrepresentation in the initial sample for this project, which was rapid and exploratory in nature, we also appreciate and recognise how understandings, perceptions and practices of solidarity might vary and be reflected as such across different socio-economic groups, communities, and regions in a highly diverse and devolved state such as the United Kingdom, and that this diversity further represents a salient point of future research in the study of solidarity.

In presenting these findings, we also acknowledge potential limitations of work given the relatively small sample size of research participants and implications for generalisability of experiences and perspectives during this time. Significantly however, the size of the research sample enabled a rapid collection of real-time data experienced by the research participants as events occurred, during a period of significant crisis in the United Kingdom, providing new research findings and perspectives on practices of solidarity which are thick in detail, verbatim, and insight. Further still, key findings from this study, namely the importance of institutionalised solidarity which emerged from the research participants also resonate with data findings from other European countries during the onset of the COVID-19 pandemic (SolPan Publications, 2022).

## Solidaristic and Non-Solidaristic Practices

### Solidarity in Practice: Proximity, the National Health Service (NHS) and Vulnerable Groups

More than half of our respondents reported experiences that we classified as solidarity according to the criteria listed above. This includes instances where people enacted or received solidarity or witnessed others doing so. These experiences can be grouped in three main categories: neighbourhoods, the NHS, and vulnerable groups. First, people referred to the strengthening of existing ‘neighbourly’ practices or starting of new ones. Neighbourly practices consisted of the provision of emotional and practical support in one’s local community. In the words of one participant:

I have an 83-year-old friend I take food to, as well, occasionally, and I keep an eye on her. She phones me up every day, and I just check if she needs anything. She’s scared to go out, she will not go out. She hasn’t gone out since the whole lockdown started, so that’s three weeks she’s been in her house. So, I do this, and I help this lady friend of mine, who’s stuck. (BG03)

Strengthening neighbourly practices of support meant being in touch with people and supporting them by providing them information, or simply by talking to them and see how they are doing. Networks of support included friends, neighbours and acquaintances but also extended to people our respondents did not have previous contact with. Support was initially ad hoc, and spontaneous practices that became more structured with time. Newly created systems of support included *WhatsApp* groups of neighbours to check on how others were doing, or to share the ordering of groceries or pharmacy needs. As one participant put it:

So, they had this idea of organising and setting up a WhatsApp group first with all the people on the street that wanted to join. And I think their very first step was to distribute red and green cards to put on the window just so that people who were in need could have put a red card. And so that whoever was available could pop down, and knock at the door, see what was wrong, see if there was anything that could be done to help, and stuff like that. (BG04)

Second, participants reported practices aimed at supporting the National Health Service (NHS) and healthcare workers that went beyond the mandatory measures of social distancing, staying at home and washing hands prescribed at the time by the government. For example, one participant explained that they avoided buying PPE equipment despite the fact that they wanted to protect themselves when going out because ‘I wouldn’t want to have detracted from supplies being available for NHS and key workers’ (BQ02). Others indicated that they chose to not use healthcare services at all, to avoid putting strain on the system:

I would say I would definitely fall into the category of people who would avoid going to the doctor, or to A&E, or anything, because of what’s going on. I would definitely be more reluctant to seek care and help, without a doubt. Not necessarily out of fear about what might happen, but more of a case of they’ve got bigger problems to deal with, kind of thing. (HT04)

In some cases, people actively responded to requests for material help by purchasing or making essential items for healthcare workers and hospitals, including masks and other care packages:

I’ve also done a little bit of shopping for hospital wards that have asked for things like toothpaste, underwear and soap, and things like that. So, I’ve bought those things in bulk and taken them off to the hospital wards. (TS01)

Third, people spoke about volunteering in more structured organisations to support vulnerable groups of people. This included participants who were already working in volunteering organisations that reorganized their activities during lockdown. In the words of one of our respondents, ‘normally I volunteer with a group for the elderly, so say over the age of 75, and we organise some tea parties for them once a month. We’re a group that come together, the same group, I think they’re all very vulnerable, we do still keep in touch with them by telephone once a week’ (BG05).

Beyond already existing volunteering organisations, new Mutual Aid groups were created during the lockdown:

So primarily what I do is there’s a community pharmacy there, which is absolutely fantastic. They know me really well. And one of the major issues that they have is that there’s quite a lot of older people and people with significant disabilities in the area. […] So, they’ve organised now with the Mutual Aid group that, so I will go in there twice a week, pick up a big pack of medication and deliver to about probably about 15 addresses. Knock on the door, obviously step back, and maybe have a bit of a chat about whatever with the person that’s inside. Because many of them are having to self-isolate for 12 weeks. (BQ01)

These forms of support were framed as contributions to institutionalised forms of solidarity, of which the NHS was seen as the most important manifestation. As these quotes illustrate, also participation in (and, at times, initiatives for) other structures of more permanent support were reported by our participants.

### Underlying Rationales Associated With Solidaristic and Non-Solidaristic Practices

#### Rule observance as a sign of care and solidarity

For many of our participants, solidaristic behaviour was closely related to obeying the rules for pandemic containment. For most, obeying rules was not, however, an end in itself, but it expressed respect and care about others. A quote from another respondent demonstrates an additional dimension of rules whose observance was considered a sign of care for others:

On Facebook I saw a little film clip that I think was from a young doctor in The Czech Republic. And she was wearing a mask. And she said wearing a mask will not protect you, but if you wear a mask it might protect me, and if I wear a mask it might protect you. So, I think in that country they were moving towards everybody wearing masks in public places, and she said just remember it may not protect you, but it will protect somebody else. And I thought that was quite impactful, and I thought yes, if we were in a crowded place, we should think about that. (HT09)

This respondent alluded to the importance of everyone complying with the rule—for example, everyone wearing masks—for the measure to be effective. In other words, for mask wearing to be seen as a sign of care, it needed to be mutual.

Reciprocity also featured in our respondents’ accounts: People were particularly willing to restrict their own freedoms and accept inconveniences—such as mask wearing, staying at home, but also volunteer work—to support those that they felt were also making (even bigger) sacrifices to help others. The paradigmatic case of people who deserved such support were key workers, and in particular, healthcare workers—which, again, represent institutionalised solidarity. In the words of one respondent,

I didn’t go out and buy PPE equipment as a result of the coronavirus because I wouldn’t want to have detracted from supplies being available for NHS and key workers. (BQ02)

### Mixing of Self and Other-Regarding Concerns

Several of our respondents suggested that the allegedly inconsiderate and non-solidaristic behaviour of others reflected a general societal shift to individualism. As one respondent put it:

It’s quite sad, really. It’s quite sad that people can be selfish, to be quite honest. It never used to be a country that was selfish, where you’d look after your neighbours and what have you, but slowly, over the decades, that’s gone, and it’s like it’s just me now. Me, me, me, me, me. I don’t care about anybody else. But I’d like to think that in this time, at the moment, people are thinking, I’m going to keep an eye on my neighbour, the elderly, look after them. But you can just see it slowly going back to being, they’re all right now. I can look after myself now. (TN01)

More commonly, however, motivations for non-solidaristic behaviour were explained by fear of COVID-19 exposure or infection, or risks to one’s psychological health. This respondent, for example, explained why she did not provide the support to a neighbour who asked to watch her children so that she could buy groceries:

She’s a single parent as well. She wanted me to have her children for an hour while she could go shopping. And I was talking to my sister about it and I thought I don’t know. I don’t know. My sister was just like it’s up to you, but I wouldn’t. It’s a risk, and it’s not worth it. She can do online shopping like everyone else, or you can put some of her things in your basket to buy for her. And I said no to her. But it was very difficult to do that because she’s a friend and we want to help her out, and we’re in the same position. (HT08)

Another respondent explained their inability to participate in neighbourhood support programmes because of their concern for their own health which could become a burden to others:

I do feel a sense of frustration because, actually, because I like people and I’m curious about people, I would, ordinarily, if I didn’t have the diabetes that I do, probably... Well, there, certainly, would be one of those wanting to get out there and do things, whether it’s [to] take stuff to people, or shopping, all the rest of it. But I just feel that I’m a bit of a risk to them, potentially, though that’s quite low. But there is a risk to me. And so, I’m thinking, well, as much as I want to, I really shouldn’t because if I become ill then that’s just another burden. (TS04)

Where participants reported that they themselves had broken rules, it was often because they perceived a certain ‘flexibility’ to the rules, or because they saw the rules as unnecessarily strict. What may have seemed as careless or non-solidaristic behaviour to the observer could, from this perspective, be better described as the conviction that the health risk was not all that serious—as illustrated in the next quote:

But that was the week leading up to that, where they’d said, avoid restaurants and pubs, if possible. Well, I didn’t really avoid them, I didn’t take that… It wasn’t a strong enough message, at first, I don’t think, to make me stay in. But once they closed the schools, they closed the restaurants and the pubs, then it was, oh, okay, all right. This is serious. (HT06)

As illustrated by these quotes—and articulated also in countless others—the considerations that led to ‘non-solidaristic’ behaviours were not always motivated only by self-interested considerations. Often, they were, indirectly or even directly, also motivated by concern for others or by concerns of furthering the pandemic.

### A Shift Towards Collective Interests: We All Depend on One Another

A small number of participants referred to the pandemic as a time of ‘collectiveness’, illustrated by the words of this respondent, for example:

*Respondent*: ‘We will see some people cooperating quite well as a society, maintaining distancing. But then, equally, you’ll go into shops and there are people who appear to have no regard for it at all’.

*Interviewer*: ‘What do you think about that when you encounter people not respecting distancing, for instance? How does that make you...? What kinds of emotions or thoughts to you have when you are put in that situation?’

*Respondent*: ‘It makes me think that people are quite rude. Because even if you don’t agree with it, this is, probably, the most collective and public issue that we’ve seen, ever. So, even if you don’t necessarily agree with stuff, you will be fully aware that this is what the world is doing. And, at least, at the moment, none of the measures put in place really make life that difficult for people when they’re out and about. It’s not that difficult to keep your space when everyone else is doing it’. (BQ02)

This respondent called the pandemic ‘the most collective issue’ that people have seen in their lifetime—and ended with a call on everyone to contribute their bit to solving it. Another respondent echoed this sentiment and added that before the pandemic, they had not been aware of how much they relied on others:

I’ve always appreciated my family and friends, but I think I didn’t realise how much I relied on other people. So, I guess in a way it’s a good thing for me to stand on my own two feet and know that I can do it on my own. And although I want them there and need them if I can’t have them, then I know that I am enough for me and my children to get on with life. (HT08)

Although this person noted that the pandemic made them realise how much they depended on others, this dependence did not seem to make them feel weak or deficient. On the contrary, it seems to have given them the knowledge that they are able to look after themselves and their family if they have to, but that life is better with the support of others.

Such support of others, however, was considered a requirement by many of our respondents to make the pandemic measures work. Some articulated a sense of frustration and also anger about the fact that it was not enough for them to behave responsibly at an individual level; instead, it required society as a whole to tackle the pandemic. ‘It makes me angry’, one person told us, ‘because, if there’s another spike, we’re all going to suffer’ (BQ01).

At the same time, even among those respondents who complained about others not observing rules, or showing a lack of care towards the others, there was usually the sense that the proportion of rule-breakers was relatively small:

I think the way I am, and I behave, and I would treat people, I would expect them really to treat it the same. So, I think on the whole most people have pleasantly surprised me. And back to this, most people in the world are good, decent, honest people. But there’s always an element of society that won’t comply. Think the rules don’t apply to them. Either because they’ve got too much money or they’re arrogant or they’re not educated or they’re young and feel they’re immune to it all or the rules don’t apply to them. (TH02)

The not complying people are perceived as people who feel different and removed from the community.

## Regretting the Absence of Solidarity: Scarce Resources, Financial Gains (or Losses), Uncertainty and Individualism

As was further evidenced in the data, some respondents also addressed a perceived lack of mutual support and solidarity. They either told us about instances where they themselves did not provide support or spoke about others who behaved in non-solidaristic ways. Broadly, reported non-solidaristic behaviours fell into three categories: Competition for (what was perceived as) scarce goods; practices related to businesses and their financial losses or gains; and breaking pandemic rules due to uncertainty around their applicability. We take each in turn.

First, many participants’ ideas of scarcity revolved around food or consumer goods and their purchasing. They related the panic buying, stock piling and hoarding that left shelves in supermarkets empty. Some participants discussed their own stockpiling, with one participant reporting also shopping for family members for fear of a future scarcity of goods:

So, we thought wow, this might really be going to happen. So, we did go and fill up our box, filled up the freezer, and filled up the fridge, and bought nappies and things, because I’ve got a daughter who’s got a baby in nappies, just in case they became difficult to get. (HT09)

Others mentioned witnessing stockpiling, as exemplified in this quote:

I think that people took more than they actually needed, and I think the horror is when they go and buy it just to make money out of it by selling it on. I think it’s awful. This is a national crisis here, and to try and see it as an opportunity to make money at other people’s expense. (TH01)

This respondent said they could understand why some people were stockpiling out of fear. However, they and others drew a moral line where people tried to profit from stockpiling financially, with one respondent likening the latter to the ‘black market in the war’: ‘These people buying all the blooming toilet roll, and then standing on the sides of the roads, selling them. That was just appalling’ (HT01).

Third, some participants spoke about others’ (or even their own) non-adherence to government regulation and advice, including taking more than the allotted one-hour of exercise per day, house parties, and socialising in groups in parks as well as seeing people travel longer distances by car to take the one-hour of exercise:

There have been cars parked outside the pub at the end of my road where people have driven to their one-day exercise, which is not allowed. And just because it’s a nice bit of scenery in the woods it doesn’t matter. They’ve been actually driving up, which I think is ridiculous. They don’t need to get in their cars, drive somewhere and then walk. (TH03)

## Calls for Greater Institutionalised Solidarity

Our respondents’ references to the lack of solidaristic practices was often intertwined with their hopes or calls for more institutionalised solidarity. Solidaristic institutions are arrangements to which people contribute according to their ability and they receive support according to their need. The NHS is the paradigmatic example of institutionalised solidarity—and our interviewees’ support for it has been discussed already. But also, other arrangements that support people who need social, economic, or psychological support are instances of institutionalised solidarity. In our interviews, there were a number of instances where people articulated the need for more institutionalised support for others in need, such as this person (SM01):

They said that the government are going to put something in place for them, but they’re not going to do anything about it until June. Which is ridiculous, really. They’ve still got to feed their children and pay their mortgages and things, but how? They’ve got no money coming in.

Another participant (SH04) gave the example of the governments bailing out big banks, but not people—and suggesting that big banks did not keep their end of the implicit social solidarity contract:

They take taxpayers’ money and […] bail out these big banks, but where are these big banks when the taxpayers need them? I don’t know, I’m just asking, because I don’t know. I don’t think the big banks are doing, I think they’re probably looking for empathy as well. […] And money shouldn’t even be a problem, my philosophy of the world is different. Because I don’t think money should be a problem for anything when it comes to saving lives, they shouldn’t be thinking about money.

Stop talking about money when it comes to a project to save lives. Like everybody needs to come together in other words. This is a worldwide matter, it’s not just one country, so money shouldn’t be an issue.

Several of our respondents highlighted that individual solidaristic actions could only happen with institutional support—specifically, the support of authorities. Next to social, economic and financial support, this also included the stringent enforcement of rules. In the words of this respondent,

[The authorities are] being a bit too easy on the ones that are flouting… They’re not taking it seriously. They’re all still going out socialising in parks and things. They’re being a bit too soft. They need to really start clamping down a bit harder on that. We’re all doing our bit, but then you’re getting certain few that are like, that’s not going to affect me. I’m going to flout it and I don’t care. They’re letting them get away with it. (TN01)

Further still, some people mentioned that the pandemic had made it clear to them how important institutionalised solidarity was, and that they wanted to contribute to it in the future (SM02):

So, all of the local bakeries, and butchers, and farm shops and all that sort of stuff are all doing home deliveries. But in the village as well, there’s a little charity called Community Count. So, there’s a residential home in the village, and that residential home is making extra meals, and members of the community are taking hot meals out to vulnerable people, and they’re also taking those vulnerable people to appointments and things like that. So, there is a lot of stuff going on, which I wanted to… I thought, when I’ve retired, I’ll go and do this stuff for a bit, before I get a job, but I daren’t go and do it. I’m frightened to go, and do it because I’m frightened of catching it.

Although there is inevitably often a gap between people’s stated intentions and their actual practice, there was still a clear sense among many of our interviewees that the pandemic made them feel—for some of them, for the first time—how important it was not only to have or provide ad hoc support to others, but have institutions in place that ensure that people are safe, economically, politically, and socially, during both periods of emergency and non-emergency.

## Discussion

While we have described some specific instances of solidaristic and non-solidaristic practices, in general the boundaries around solidaristic actions were fluid as revealed in our key findings. As we engaged with the ways that solidarity emerged in the interview data, it became clear that it is difficult to draw clear lines around what is, and is not, solidaristic practice during this period of national emergency in the United Kingdom. Even seemingly self-interested motivations, such as wanting to avoid COVID-19 exposure and infection, often blended with more collective concerns, such as not wanting to be a burden on the healthcare system. This resonates with understandings of solidarity that see it as a practice comprising both self- and other-directed elements (e.g. Dean 1996; Prainsack and Buyx 2017; Atuire and Hassoun 2023). Even where solidaristic practices could be identified, at this early stage in the pandemic, they were often local. This was even though many participants identified a shifting mindset in terms of mutual respect, responsibility, and inter-dependency. In a way, solidarity was most prominent and expressed where people felt its absence: Where they expected and hoped for solidarity from others and it was not there, solidarity took on a very prominent place in people’s narratives and experiences during lockdown.

Participants also referred to the collective nature of risks and of achieving benefits. In a society where many people grew up with the belief that they were individually responsible for controlling their own risks—by buying insurance, by living healthily, by getting a good education to protect themselves from the risk of unemployment and poverty. It seemed to be difficult for some people to accept that their own risks could only be mitigated by collective action, particularly during periods of emergency. Some of our respondents voiced frustration and even anger about the fact that their own behaviour was not sufficient to avoid infection but that it required everyone to chip in. Rule observance during the pandemic, in turn, was seen by many of our respondents as a sign of care and solidarity—and the ignoring and breaking of rules as the opposite. Various reasons were given for the latter: That there are two kinds of people, the rule observant ones, and the rest; that people had generally become more selfish in recent years, or that some people were simply selfish ‘by nature’. At the same time—and often referring to instances in which they themselves had broken a rule—many also stated that it was not always possible to comply with all measures. Others who broke rules did not consider this as endangering the health of others. It was also acknowledged that being able to follow all rules required a quite privileged position in society.

Some of our participants voiced a need for greater and more concerted political, social and other support and infrastructure to enable people to enact solidarity at individual levels. First, we found that existing institutional, political, and cultural infrastructure supports effective action at individual and group levels. By cultural we mean that the NHS, for example, is strongly culturally embedded in all countries of the United Kingdom. Respondents understand its purpose, its individual and collective benefits, and it has strong ties to national identity. In this way, people can easily conceptually understand its importance and place in their lives, and the pandemic. They found it easy to manifest their support for the NHS, and to adapt their practices in support of it. Second, institutions also need to provide practical—social, economic and psychological—support in a health crisis, not only to get through the crisis as well as possible, but also to be able to enact or sustain solidarity at individual levels. Constant calls by media and political leaders for citizens to enact solidarity without being seen to support solidarity in any way they can—by supporting the most vulnerable, or by working towards vaccine equity in a global context—are likely to cause more harm than good (EGE 2022). Third, some of our respondents felt that, where there are high stakes in solidaristic practices, meaning that a large number of ‘free riders’ would cause harm to others, institutions need to support solidaristic practice by enforcing it. In cases where physical or grave financial or psychological harm may result from too many people not adhering to solidaristic policies (e.g. keeping a physical distance to others, or wearing masks in crowded places), participants looked to the state to police solidaristic behaviour and to penalise those who transgressed.

These empirical findings regarding the manifestation of solidarity in real word settings both advance the theoretical understanding of solidarity and suggest emergent, yet practical considerations as to how solidarity can be better supported and utilised during public health episodes. Before we discuss the latter, we examine how the practices of solidarity that our participants reported map onto existing theoretical understanding of solidarity.

As noted, Prainsack and Buyx’ (2017) conceptualisation of solidarity resonates with our findings in the sense that solidarity is a practice that includes both self-interested and other-regarding aspects that often shade into one another. Furthermore, these authors offer a typology of solidaristic practice along three different tiers, which we find helpful in differentiating more ad hoc and singular from more ‘stable’ and systemic forms of solidarity. As noted, Prainsack and Buyx (2017) differentiate between solidarity enacted from person to person (tier 1), at the level of groups (tier 2) and the level of institutions (tier 3). In our data, the practices of people who supported others that they had not previously been in contact with, such as elderly neighbours, illustrates how the pandemic situation served as a new commonality that people saw between themselves and others with whom they previously did not interact with much. Particularly the strengthening of practices of neighbourly interaction can be seen as instances of interpersonal, tier 1 solidarity. New support networks—as more institutionalised forms of the ad hoc and often one-to-one support represented by interpersonal solidarity—are also instances of group-level (tier 2) solidarity. Finally, a third of our respondents explicitly mentioned the NHS and the people working within it in need of support and protection. This is a paradigmatic example of institutionalised (tier 3) solidarity, which has arguably received more attention during the pandemic in many societies across the globe (Prainsack, 2022).

At the same time, some of our data cast doubt in certain areas about the ‘fit’ of Prainsack and Buyx’s definition of solidarity as ‘an enacted commitment to carry “costs” (financial, social, emotional or otherwise) to assist others with whom a person or persons recognise similarity in a relevant respect’ (Prainsack and Buyx, 2012:52). We found that costs were often difficult to identify. In the interview data, at times it was difficult to determine precisely what the ‘cost’ of a given action was for an individual. Some actions—for example, the practice of public clapping for the NHS—arguably incur no significant cost for participants (besides the time taken) but are intended as an act of public appreciation and recognition of the sacrifice of others. Such practices cross across the interpersonal, tier 1 of solidarity and tier 3 of institutionalised solidarity—in the sense that clapping for key workers could be seen to an expression of support for more permanent institutions and policies of solidarity (besides other such expressions of support).

In this context, also the approach to solidarity put forward by Dawson and Jennings which reject that ‘costs’ are a necessary requirement for solidarity (Dawson and Jennings, 2012) is particularly relevant for our work. They ‘hold solidarity to be a deep and enmeshed concept, a value that supports and structures the way we in fact do and ought to see other kinds of moral considerations’ (Dawson and Jennings, 2012: 73). This idea of solidarity as a value, was expressed in our data—‘the foundational aspect of solidarity can be captured by the fundamental idea of “standing up beside”’ (Dawson and Jennings, 2012: 74). This was usually expressed in concern for others, and small activities. Similar to Prainsack and Buyx (2017), who emphasise that although both self- and other-regarding concerns are usually at work in solidaristic practice, personal gain cannot be the primary motivation, Dawson and Jennings also argue that it is important that solidaristic action does not derive out of expectation of benefit from the other, but out of moral concern for that other. In our data we found that motivations for ‘solidaristic’ practice were often mixed, and that respondents were often conscious of the impact of collective action on their personal risk—but also the other way round.

Our data also supports the warning of authors writing solidarity in the context of previous epidemics and pandemics that the sustainability and power of person-to-person solidarity in acute health crises should not be overestimated (e.g. Baum et al. 2009; Determann et al. 2016). Our data seem to support Prainsack and Buyx’ argument in 2011 that in pandemics, because risks are unevenly distributed and stakes are so high, ‘it is unreasonable to expect that entire populations [ … ] will accept the costs of containing pandemics out of solidarity with each other’ (Prainsack and Buyx, 2011: 68).

Our data also points to new practical ethical questions around the institutionalisation of ‘solidarity’. While policy makers mobilising solidarity in times of health crises may have individual behaviour in mind, we found that people find institutionalised forms of solidarity equally important—some even see it as a precondition for individual solidarity to emerge and remain strong. If individual solidarity is ‘not enough’ then what shape should institutional solidarity take? One possible answer to this is to strengthen solidaristic institutions that already exist, but that were harmed by underfunding and privatisation in recent years, such as the NHS. Austerity politics, then, becomes an ethical concern. Another possible answer is to point to the importance of ensuring that ‘free riding’ within solidaristic institutions is recognised as an important factor that decreases trust in the institution in itself. This does not mean that everyone has to do or contribute the same, which runs counter to the very essence of a solidaristic institution—which can be defined as one where people contribute according to their ability and from which they receive support according to their need. It does, however, mean that people’s contribution according to their ability is taken seriously as a moral (and, where applicable, a legal) requirement. At the same time, however, more empirical and conceptual research is needed to delineate where such ‘enforced solidarity’ at the level of institutions is ethically permissible or even required, and where it runs the risk of creating problematic exclusions.

## Conclusion

While our data maps onto some key conceptualisations of solidarity relevant to public health, we find that even these conceptualisations fall short in capturing the breadth of practices pertaining to solidarity in this current pandemic. Besides the problem of delineating what counted as a solidaristic practice and what did not (e.g. does clapping for healthcare workers qualify? How do we classify complaints about the lack of support for certain groups?), existing conceptualisations of solidarity do not fully capture the fluidity of practices and perceptions of and around solidarity. For example, we found that people’s descriptions of their own practices meandered from enacting support for others at significant cost for themselves (e.g. by refraining to use healthcare services) to refusing to provide support for a neighbour out of concern for one’s own safety, or even out of frustration that some ‘don’t play by the rules’. Most current conceptualisations of solidarity look at solidarity in very specific contexts where solidaristic practices and institutions are relatively stable. During the onset of COVID-19, the speed and scale of the pandemic rapidly increased both public and scholarly interest in solidaristic practices across local, national, and global levels. This was driven in large part by government and media rhetoric and application of the concept yet with very little understanding into how individuals and populations experienced or perceived solidaristic practices or a lack thereof within their communities and from institutions. The data collected and analysed during these interviews during the first national lockdown in the United Kingdom therefore reflect a range of understandings and perceptions of solidaristic practice, as well as the blurred boundaries between solidarity and non-solidarity collected in real-time and during both a national and global period of instability.

In addition, for scholarship on solidarity to be of greater use in the context of public health (and health crises in particular), it needs to pay greater attention to institutional forms of solidarity. Again, here we reflect how one of the most significant emergent findings from our data was the extent to which our respondents called for political and institutional infrastructures that implement or support greater solidaristic practices (in the forms of political, economic and social protection) from the top down. Key within the United Kingdom, the NHS is one such structure—social and economic support systems are further examples which were underscored. Participants in our study also articulated the need for different forms of authority to support, facilitate, uphold, and reward the solidaristic practices carried out at the local and institutional level.

## Footnotes

1 We acknowledge the contributions of Dr Katharina Kieslich to reviewing this literature.
